# Critical neuroscience—or critical science? A perspective on the perceived normative significance of neuroscience

**DOI:** 10.3389/fnhum.2014.00336

**Published:** 2014-05-20

**Authors:** Stephan Schleim

**Affiliations:** ^1^Theory and History of Psychology, Heymans Institute for Psychological Research, Faculty of Behavioral and Social Sciences, University of GroningenGroningen, Netherlands; ^2^Research Center for Neurophilosophy and Ethics of Neurosciences, Munich Center for Neurosciences, Ludwig-Maximilians-University MunichMunich, Germany

**Keywords:** neuroethics, cognitive enhancement, moral decision-making, forensic neuroscience, science communication

## Abstract

Members of the *Critical Neuroscience* initiative raised the question whether the perceived normative significance of neuroscience is justified by the discipline’s actual possibilities. In this paper I show how brain research was assigned the ultimate political, social, and moral authority by some leading researchers who suggested that neuroscientists should change their research priorities, promising solutions to social challenges in order to increase research funds. Discussing the two examples of cognitive enhancement and the neuroscience of (im)moral behavior I argue that there is indeed a gap between promises and expectations on the one hand and knowledge and applications on the other. However it would be premature to generalize this to the neurosciences at large, whose knowledge-producing, innovative, and economic potentials have just recently been confirmed by political and scientific decision-makers with the financial support for the *Human Brain Project* and the *BRAIN Initiative*. Finally, I discuss two explanations for the analyzed communication patterns and argue why *Critical Neuroscience* is necessary, but not sufficient. A more general *Critical Science* movement is required to improve the scientific incentive system.

“Ideologies, philosophies, religious doctrines, world-models, value systems, and the like will stand or fall depending on the kinds of answers that brain research eventually reveals. It all comes together in the brain” (Sperry, 1981: 4).

In the year Roger W. Sperry received the Nobel Prize for his research on the functional specialization of the cerebral hemispheres, he expressed worries that the public had lost trust in science, including his own field: neuroscience. Indeed, an editorial in *Science* written by the President (on leave) of the New York University and Deputy Secretary of Energy John C. Sawhill warned of the negative consequences of a “public disillusionment”, “credibility gap”, and “growing cynicism” about science (Sawhill, 1979). In Sperry’s view, neuroscientists should change their research priorities and emphasize the possible social benefits of their enterprise; this should be easy, he argued, because all political, social, and philosophical matters were ultimately subjects of brain research. This shift should allow a fusion of science and religion, the descriptive and the prescriptive, to overcome global problems caused by increasing population, pollution, poverty, and energy demands (Sperry, 1981). Twenty-five years later his former PhD student Michael S. Gazzaniga would revive this vision in the introduction of his book “The Ethical Brain” (Gazzaniga, 2005; see also Schleim, 2005; Schleim and Schirmann, 2011).

In the presidential declaration heralding the “Decade of the Brain” 10 years after Sperry voiced his concerns, the broad social potential of the neurosciences was explained with respect to neurodegenerative diseases as well as the “War on Drugs”. President George H. W. Bush called “upon all public officials and the people of the United States to observe that decade with appropriate programs, ceremonies, and activities” (Bush, 1990). The Commission of the European Communities soon followed with a similar declaration heralding the “European decade of brain research” (Pandolfi, 1993). Sperry’s fears that the neurosciences might suffer budget cuts were thus overcome in the 1990s. In spite of recent investigations of science communication reporting a raise in “neuroskepticism” in scholarly as well as public reports on neuroimaging (Rachul and Zarzeczny, 2012; Whiteley, 2012) and broader analyses into “neuromythology” (Hasler, 2012; Satel and Lilienfeld, 2013), the political-scientific decisions in 2013 to fund the major research endeavors *Human Brain Project* and *BRAIN Initiative* emphasized that the neurosciences still enjoy high public confidence in their knowledge-producing capacities as well as their potential to drive technological innovation and, not the least, economic growth.

*Critical Neuroscience* is a young initiative to probe “the extent to which discussion of neuroscience … matches the achievements and potential of neuroscience itself”.^1^ By means of critique and including interdisciplinary perspectives, its members try to better understand the knowledge-producing and -communication processes in the neurosciences and its wider ramifications (Choudhury et al., 2009; Slaby and Choudhury, 2012). Notably, Jan Slaby asked: “Does neuroscience indeed have such wide-ranging effects or are we collectively overestimating its impacts at the expense of other important drivers of social and cultural change?” (Slaby, 2010: 397). In this paper, I follow a two-fold strategy to propose a preliminary answer to his question: first, I skeptically^2^ discuss two examples of neuroscience technologies that would obviously have strong social/normative implications when applied, namely (1) pharmacological enhancement and (2) (im)moral neuroscience; second, I relate Critical Neuroscience to (3) recent critical analyses of the general scientific knowledge-making and -communication processes.

## Promises of pharmacological enhancement

That healthy people use psychoactive substances to influence their experiences or relations with other people and society was not new in the early 2000s: besides references to ancient cultural or religious practices, the medical-anthropological investigation of what Nicolas Rasmussen called “America’s First Amphetamine Epidemic 1929–1971” provided clear evidence for this (Rasmussen, 2008a, b). New was framing the phenomenon in terms of “cognitive enhancement”.^3^ Indeed, also the President’s Council on Bioethics’s related report “Beyond Therapy: Biotechnology and the Pursuit of Happiness” published at the beginning of that decade discussed superior performance exclusively with respect to sports and muscle enhancement (Bioethics Council, 2003). When addressing the possibility of stimulant drugs as behavior improvement, they strongly emphasized social-moral over cognitive alterations, namely, children’s “ability and willingness to be considerate, show respect, pay attention, carry out assignments, accept responsibility, deal with stress and disappointment, and practice self-control” (Bioethics Council, 2003: 71). At that time, the drugs that would later be discussed as possible cognitive enhancement substances by neuroethicists had already been known for years (modafinil) to decades (amphetamine, methylphenidate).

The sudden emphasis on cognitive performance by scientific researchers, particularly performance-enhancing behavior of students (Farah et al., 2004; Greely et al., 2008), coincided with political initiatives to define a nation’s value in terms of its citizen’s “mental capital” (Beddington et al., 2008; Foresight, 2008; see also Slaby, 2010). Obviously, the science underlying the “mental wealth of nations” (Beddington et al., 2008), that is, cognitive neuroscience, became of utmost public importance, because “if we are to prosper and thrive in our changing society and in an increasingly interconnected and competitive world, both our mental and material resources will be vital” (Foresight, 2008: 9). Notably, the central claim that students *increasingly* consumed stimulant drugs to become better could not be confirmed so far (Smith and Farah, 2011). It should be considered, though, that many of the prevalence surveys define enhancement as mutually exclusive with treatment, excluding consumption as soon as drugs are medically prescribed. This neglects the possible interpretation that there actually is an increase in stimulant consumption due to a medicalization of normal or slightly sub-threshold cognitive performance (Rubin, 2004; Abraham, 2010).^4^

While stimulant drugs may improve performance of healthy people in some cognitive tests under laboratory conditions (Repantis et al., 2010; Smith and Farah, 2011), their benefit in real-life settings is much less clear. Pharmacologists like Boris B. Quednow provided theoretical arguments for why we should not expect too much given the current knowledge of brain function (Quednow, 2010). For example, just increasing neurotransmitter levels beyond normal levels is likely to decrease performance. Quednow’s suggestion to call stimulants “secondary enhancers” because their effects on cognition seem to be (at least partially) mediated by enhancing motivation—how people feel about performance and pressure—is supported by a recent survey of stimulant consumers’ self-descriptions at an “elite university” in the USA that identified four *emotional* patterns: feeling up, drivenness, interestedness, and enjoyment (Vrecko, 2013). Thus, “cognitive enhancement” may not be that cognitive, after all.

Even more sobering are two broader observations: first, that many pharmaceutical companies decided to close their psychiatric laboratories because further efforts do not seem to justify the expected benefits (Amara et al., 2011; van Gerven and Cohen, 2011); second, that even in cases of diagnosed ADHD—thus in the presence of a therapeutic behavioral-cognitive target—the prescription of stimulants does not seem to make a lasting difference to performance (Currie et al., 2013; Sharpe, 2014). If the prospects of pharmacological research in these therapeutic contexts are poor, after decades of intensive research and in the light of developed clinical models, the speculations on the effects in healthy people seem even more preposterous.

Disturbingly, a recent communication analysis found evidence that the media are biased towards positive representations of pharmacological enhancement and an exaggeration of its prevalence (Partridge et al., 2011). In line with this research, anecdotal evidence suggested that scholarly publications by scientific experts themselves underlie this communication pattern (Schleim, 2010). This raises the suspicion that the debate keeps reinforcing itself while lacking strong evidence that the expectations can be met in the near future. From a communication perspective it can be a successful attention-maximizing strategy to first hype a new discovery/development and later debunk it, thus multiplying the number of reports, a pattern not uncommon with respect to pharmacological discoveries (Williams et al., 2008).

## Promises of (im)moral neuroscience

Morality in its several facets, what people are doing and why as well as what they should do, has fascinated thinkers at least since antiquity (Nadelhoffer et al., 2010). It is common to distinguish the *prescriptive*, what should be done, and the *descriptive*, what is the case. Philosophers developed arguments that it amounts to a fallacy to derive a prescription from a pure description (Moore, 1903). Initially we have seen the two statements by Sperry and Gazzaniga clearly expressing the idea that descriptive knowledge of the brain will inform us on what we should do. Accordingly, Joshua D. Greene and colleagues who confronted students with descriptions of moral dilemmas in a brain scanner suggested from the outset that their research could decide the stalemate position in which, in their view, moral philosophers had been caught for a long time (Greene et al., 2001, 2004). Their attempt was twofold, associating (1) utilitarian/consequentialist judgments favoring the benefit of the greatest number of people with “cognitive/rational” brain areas (e.g., the dorsolateral prefrontal cortex) and evolutionary recent developments; and (2) other kinds of judgments favoring people’s intentions or general principles/duties with “emotional/irrational” brain areas (e.g., the ventromedial prefrontal cortex and amygdalae) and evolutionary ancient developments (Greene et al., 2004; Greene, 2008). Indeed, these interpretations were endorsed quickly by moral philosophers arguing that ethical utilitarianism has a rational basis, while a competing deontological ethics has not (Singer, 2005).

**Figure 1:**
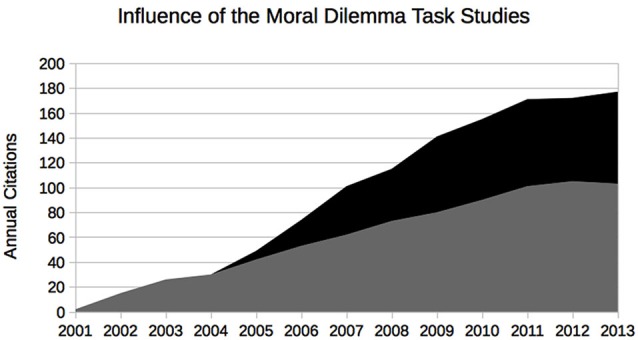
**Evidence of the communication success of the original moral neuroscience studies by Greene et al. from the ISI Web of Science**. The authors were not only the first to present difficult philosophical moral dilemmas to their subjects in the brain scanner, but suggested a strong normative significance of their findings, provoking responses from other scholars. Gray: Greene et al. (2001) (overall 782 citations); black: Greene et al. (2004) (overall 446 citations).

It goes without saying that the association with a certain brain area or a particular evolutionary stage does not in itself have any prescriptive force; however, with a rather basic assumption that a moral theory should suffice demands of rationality the argument becomes complete. Virtually all aspects of the experiment and its interpretations have been criticized by neuroscientists, psychologists, and philosophers alike (e.g., Joyce, 2008; Schleim, 2008; Berker, 2009; Kahane, 2011; Waldmann et al., 2012). However, for moral neuroscience to become a scholarly and public communication success (see Figure 1), the prescriptive/normative claims did not have to be justified; it was sufficient that they *seemed* plausible, that it seemed that science aided by brain scanners has genuinely new ways of informing or perhaps even deciding philosophical/moral debates (Schleim, forthcoming).

Generations of anthropologists and moral psychologists before had gathered evidence on the development, cognitive-emotional mechanisms, and cultural diversity of morality, but suddenly in 2001 with the publication of the first neuroimaging experiments the situation seemed to have changed. It seems fair to say that of the seven different psychological-neuroscientific theoretical accounts of morality distinguished by Jorge Moll et al., all the evidence gathered hitherto does not unequivocally favor any particular one (Moll et al., 2005). While the science communication accompanying the original study by Greene et al. suggested the philosophical relevance of the research, even putting forward the idea that the new findings could make moral philosophers superfluous (Helmuth, 2001), so far the opposite has been the case: theoreticians of all kinds responded to the prescriptive/normative claims and emphasized how these neuroscientific reports rely on theoretical presumptions and individual interpretation.

While theoretical in its scope, moral neuroscience is used to provide the ultimate answers of human right and wrong that Sperry and Gazzaniga called for. More applied/technical implications are promised by the complementary research that might be coined “immoral neuroscience”: the investigation of what makes us behave immorally or criminally. The historian Peter Becker suggested that criminal behavior was an essential aspect of biological/neuroscientific accounts of human beings to emphasize their social relevance (Becker, 2012). Indeed, journals like *Nature* feature detailed reports when neuroscientists provide expert testimony in criminal courts (e.g., Hughes, 2010).^5^

Recently, Adrian Raine coined the notion that criminals have “broken brains, brains that are physically different from those of the rest of us” (Raine, 2013: 180). Based on the present findings of (im)moral neuroscience and an optimistic evaluation of future discoveries, he predicts the development of a new biopsychological screening and intervening program incorporating extended forms of preventive detention for the sake of public safety within the next 20–30 years, a proposal certainly calling for more critical scrutiny elsewhere (Rose, 2010).

## Conclusion and outlook

With respect to the question raised by Jan Slaby, the examples of cognitive enhancement and (im)moral neuroscience strongly suggest that notwithstanding the influences of neuro-collaborations and related funding schemes within academia, we scholars indeed collectively overestimate the practical and translational social impact of this research so far. The communicated promises as well as the scholarly and public attention given to these possibilities are, in my view, in no way justified by the scientific possibilities. It goes without saying that this tentative conclusion cannot readily be generalized to different examples or initiatives like the *Human Brain Project* or the *BRAIN Initiative*, although they are likely to be driven by the same incentive structures. This emphasizes the importance to raise considerable concerns with respect to neuroscience communication. In the remainder of this paper I will thus present two explanations of the found communication patterns and suggest improvements:

First, particularly with respect to the possibilities and limitations of functional Magnetic Resonance Imaging (fMRI) as a research tool we could witness exaggerated expectations regarding knowledge about the functioning of the human mind that were gradually diminished by a focus on biological, psychological, and statistical limitations (Logothetis, 2008; Vul et al., 2009; Margulies, 2011). Communication patterns in print media from 1995–2004 show clear evidence for an overwhelming optimism with respect to the technology’s possibilities (Racine et al., 2005, 2010), while for the period from 2005–2009, including online media, the presence of critical reports was emphasized (Whiteley, 2012), reflecting a trend in scientific reviews (Rachul and Zarzeczny, 2012). Felix Hasler suggested that this exemplifies a Gartner Hype Cycle where a technology trigger leads to a peak of inflated expectations, followed by a trough of disillusionment, a slope of enlightenment, and, finally, a plateau of productivity (Hasler, 2012). However, the neurosciences as a whole are so diverse and complex that this explanatory pattern should presently only be applied to single technologies such as fMRI.

Second, the exaggerated promises could be instances of strategic behavior driven by scarce means in a competitive and uncertain environment. Analyses of science communication in general emphasized publication and commercialization pressures, media practices, and public expectations, as feeding a “hype pipeline” (Bubela et al., 2009; Caulfield and Condit, 2012). These pressures may counteract basic scientific values such as honesty, doubt/skepticism, or openness (IAC/IAP, 2012; DFG, 2013). Indeed, current or past presidents of neuroscience institutions worldwide emphasized the pressure to publish in selective high-impact journals and achieve competitive research grants (Amara et al., 2011) and warned against the “corrupting force” of superficial standardized measures of scientific quality like the Impact Factor (Marder et al., 2010).

**Figure 2:**
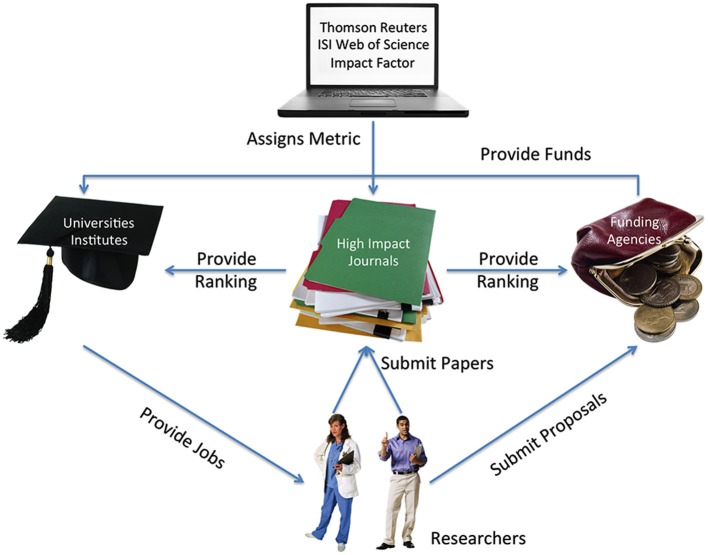
**The scientific incentive system, simplified**. Researchers compete for scarce places in high-impact science journals and limited research funds. Their access to these resources is decisive for hiring and, eventually, tenure decisions at universities or research institutes. The *ISI Impact Factor* itself is an arbitrary measure calculated by a commercial company, now owned by Thomson Reuters, representing the average number of citations of a journal’s articles in the previous 2 years, a measure originally developed for librarians. It is used by knowledge and funding institutions to assess scientists’ quality, because it seems to meet the needs of a standardized, easily comparable, and objective way of assessing research and researchers. Many scholars believe that this incentive system negatively influences decisions of scientists, particularly young scholars competing for tenured positions. Figure created using Microsoft® Clip Art. Used with permission from Microsoft (http://www.microsoft.com/.

The present situation of the scientific communication and academic incentive system (see Figure 2) actually exemplifies the behaviorists’ social engineering technology: that the environment’s reinforcement structure, the contingencies of punishment and reward, influences behavior (Skinner, 1971). Many scholars do what they perceive as a success-maximizing strategy in order to receive the rewards of public attention, research funds, the possibility to publish in high-impact journals, and tenured positions. *Critical Neuroscience* contributes to understand this reward system. However, it would be naïve to think that these forces are restricted to the neurosciences; or that scholars actively engaging in the sociology of neuroscience in general or *Critical Neuroscience* in particular are completely immune to the present pressures and rewards.

In a reflection on the “neuroscientific turn”, Melissa Littlefield and Jenell Johnson discuss the possibility that we ourselves may construct some of the risks that we subsequently propose to manage and that not only neuroscientists, but also scholars from the humanities and social sciences are under pressure to adapt to an academic world with scarce resources, favoring scientific research promising practical significance (Littlefield and Johnson, 2012). Such pressures may also affect critical scholars selecting research questions or investigating institutions where interesting things are happening to increase communication success at the cost of representativity and neutrality. Neuroethics was originally conceived to protect society from abuses of neurotechnology, but due to occupational needs and dependencies it may also shield neuroscience from society’s critique (Conrad and De Vries, 2011). It would be deplorable if *Critical Neuroscience* followed this example. It should be noted, though, that some of its members explicitly expressed awareness of such pressures and distanced themselves from neuroethics (Choudhury et al., 2009; Slaby and Choudhury, 2012).

A general *Critical Science* movement is necessary in order to change the incentive structure (Nosek et al., 2012), to guarantee scholarly autonomy and independence, and to promote behavior in accordance with basic scientific norms and values (IAC/IAP, 2012; DFG, 2013). That is, *Critical Neuroscience* is necessary; but it is not sufficient. We need to move past Sperry’s initial proposal, to work according to principles that guarantee scholarly and public trust in the long run instead of promising applications as a successful fundraising strategy in the short run at the cost of credibility. We need a *Critical Science* structure that also rewards doubt, skepticism, authenticity, honesty, and reluctance to oversimplify science or prematurely translate scientific knowledge into practice even when this is uncomfortable. I followed a two-fold strategy to answer Slaby’s question whether the neurosciences indeed have the wide-ranging effects often assumed; based on my limited analysis, both parts point in the same direction: the underlying issues go beyond the neurosciences and beyond *Critical Neuroscience*.

## Conflict of interest statement

The authors declare that the research was conducted in the absence of any commercial or financial relationships that could be construed as a potential conflict of interest.

## Footnotes

^1^See the project description on their website http://www.critical-neuroscience.org/#1 accessed February 23 2014).

^2^Note that skepticism and doubt are often considered as basic norms or values of science in official declarations of scientific bodies (IAC/IAP, 2012; DFG, 2013).

^3^An ISI Web of Science topic search for “cognitive enhancement” yields 661 hits with the first incidence in 1986; the 1990s, aggregated, have 51 incidences or 4.3 annually; the 2000s 274 or 22.9, respectively; the first 4 years of the 2010s already exceed this whole previous decade with 319 incidences or 79.8, respectively.

^4^The annual production quotas in the USA increased from 12,700 kg in 2004, when they were already on a high level, to 49,000 kg in 2014 for amphetamine and 28,693 kg to 96,750 kg for methylphenidate; that is, almost four-fold for the former and 3.4-fold for the latter (US Drug Enforcement Administration, October 2 2013).

^5^See also Nature News of October 30 2009 featuring a murder case in Italy www.nature.com/news/2009/091030/full/news.2009.1050.html) or the Nature special “Science in Court” www.nature.com/news/specials/scienceincourt/index.html), both accessed on February 23 2014.
